# Supplementary material for: The Italian Validation of the Beck Cognitive Insight Scale: Underlying Factor Structure in Psychotic Patients and the General Population
Source: Int J Environ Res Public Health. 2023 Aug 24;20(17):6634. doi: 10.3390/ijerph20176634 (PMC10487986; doi:10.3390/ijerph20176634)
Supplement: Supplementary file 1 [file ijerph-20-06634-s001.zip › ijerph-2451018-supplementary.pdf]

Table S1. Internal consistency of BCIS in GP and SZ

	GP N=624		SZ N=130	
	Cronbach's alpha	McDonald's omega	Cronbach's alpha	McDonald's omega
**BCIS 15-items**	0.501	0.222	0.575	0.435
**SR**	0.564	0.566	0.583	0.587
**SC**	0.628	0.630	0.608	0.616

GP=General population; SZ= Patients with schizophrenia; BCIS=Beck Cognitive Insight Scale; SR=Self-Reflectiveness; SC=Self-Certainty

Table S2. Inter-item correlation of the BCIS in SZ

IT=Item

	IT 1	IT 2	IT 3	IT 4	IT 5	IT 6	IT 7	IT 8	IT 9	IT 10	IT 11	IT 12	IT 13	IT 14	IT 15
**IT 1**	1														
**IT 2**	-0.031	1													
**IT 3**	0.164	-0.031	1												
**IT 4**	0.275	-0.052	0.189	1											
**IT 5**	0.205	0.005	0.114	0.151	1										
**IT 6**	0.176	-0.186	0.259	0.303	0.28	1									
**IT 7**	0.083	0.125	0.046	-0.06	-0.207	-0.043	1								
**IT 8**	-0.009	-0.222	0.047	0.034	0.221	0.295	-0.212	1							
**IT 9**	-0.022	0.139	-0.054	-0.065	0.011	-0.03	0.341	0.086	1						
**IT 10**	0.082	0.121	0.099	0.085	-0.003	0.007	0.316	0.014	0.169	1					
**IT 11**	0.307	0.046	-0.014	0.219	0.114	0.082	0.18	0.088	0.072	0.295	1				
**IT 12**	-0.104	0.053	-0.046	0.041	0.163	0.262	-0.088	0.353	0.079	-0.023	0.086	1			
**IT 13**	-0.037	0.371	0.062	-0.004	0.07	-0.061	0.211	-0.081	0.333	0.346	0.076	0.109	1		
**IT 14**	0.172	0.105	0.051	0.088	0.08	0.1	0.018	0.176	0.096	-0.026	0.135	0.25	0.072	1	
**IT 15**	0.041	-0.158	0.151	-0.008	0.105	0.064	0.019	0.085	0.097	-0.011	0.138	0.022	-0.022	0.129	1

Table S3. Inter-item correlation of the BCIS in GP

	IT 1	IT 2	IT 3	IT 4	IT 5	IT 6	IT 7	IT 8	IT 9	IT 10	IT 11	IT 12	IT 13	IT 14	IT 15
**IT 1**	1														
**IT 2**	-0.189	1													
**IT 3**	0.003	0.077	1												
**IT 4**	0.321	-0.111	0.15	1											
**IT 5**	0.225	-0.158	0.118	0.402	1										
**IT 6**	0.344	-0.207	0.08	0.252	0.413	1									
**IT 7**	-0.022	0.308	0.012	0.073	-0.028	-0.081	1								
**IT 8**	0.165	-0.094	0.032	0.095	0.119	0.258	-0.202	1							
**IT 9**	-0.011	0.261	-0.099	-0.001	-0.029	-0.118	0.278	-0.036	1						
**IT 10**	-0.015	0.176	0.028	0.049	0.069	-0.008	0.297	-0.06	0.151	1					
**IT 11**	0.108	0.007	-0.03	0.138	0.16	0.002	0.185	-0.003	0.171	0.349	1				
**IT 12**	-0.041	0.034	-0.103	-0.085	-0.054	-0.047	-0.014	0.178	0.078	-0.107	-0.099	1			
**IT 13**	-0.186	0.347	-0.028	-0.16	-0.164	-0.183	0.307	-0.093	0.317	0.157	0.033	0.16	1		
**IT 14**	0.013	0.017	-0.122	-0.045	-0.042	0.029	-0.012	0.235	0.096	-0.132	-0.052	0.39	0.134	1	
**IT 15**	0.265	-0.089	-0.039	0.249	0.281	0.197	-0.01	0.156	0.049	0.063	0.155	0.024	-0.08	0.051	1

IT=Item

Table S4. EFA with orthogonal rotation (varimax) of 6 factors solution and internal consistency of 6 factors solution in SZ

EFA= Exploratory Factor Analysis; IT=Item; The items that reach the acceptable factor loading index (≥|0.40|) are highlighted in bold

	Factors							
	1	2	3	4	5	6	Cronbach's alpha	McDonald's omega
**IT 8**	**0.648**	-0.007	0.079	-0.236	0.032	0.014	0.521	0.521
**IT 12**	**0.6**	0.029	-0.002	0.097	-0.004	0.147		
**IT 7**	-0.262	**0.657**	-0.016	0.018	0.133	0.023	0.534	0.570
**IT 9**	0.134	**0.538**	-0.07	0.122	-0.015	0.101		
**IT 10**	0.021	**0.419**	0.115	0.188	0.329	-0.368		
**IT 6**	0.351	-0.019	**0.535**	-0.173	0.041	0.03	0.500	0.511
**IT 3**	-0.034	0.085	**0.511**	-0.033	-0.043	-0.002		
**IT 4**	0.041	-0.107	**0.426**	0.008	0.274	0.015		
**IT 2**	-0.077	0.101	-0.091	**0.675**	0.031	0.12	-	-
**IT 11**	0.107	0.156	0.034	-0.003	**0.733**	0.042	0.470	0.470
**IT 1**	-0.134	-0.021	0.382	-0.041	**0.428**	0.23		
**IT 14**	0.242	0.092	0.077	0.079	0.13	**0.428**	**-**	**-**
**IT 5**	0.3	-0.129	0.314	0.072	0.117	0.075		
**IT 13**	0.11	0.416	0.077	0.561	0.006	-0.106		
**IT 15**	0.086	0.136	0.099	-0.188	0.054	0.143		

Table S5. EFA with orthogonal rotation (varimax) and internal consistency of 4 factors solutions in SZ

	Factors					
	**1**	**2**	3	4	Cronbach's alpha	McDonald's omega
**IT 7**	**0.721**	-0.263	0.025	-0.057	0.573	0.534
**IT 9**	**0.662**	0.217	-0.216	-0.026		
**IT 10**	**0.601**	-0.044	0.238	0.134		
**IT 12**	0.011	**0.766**	-0.124	0.098	0.521	0.506
**IT 8**	-0.069	**0.693**	-0.013	-0.318		
**IT 5**	-0.136	**0.473**	0.37	0.103		
**IT 14**	0.165	**0.462**	0.112	0.025		
**IT 1**	0.09	-0.06	**0.708**	-0.031	0.455	0.444
**IT 4**	-0.089	0.094	**0.686**	0.099		
**IT 3**	0.054	0.021	**0.473**	-0.118		
**IT 2**	0.253	0.021	-0.07	**0.759**	0.000	-0.373
**IT 15**	0.266	0.141	0.051	**-0.602**		
**IT 11**	0.409	0.104	0.455	-0.075		
**IT 6**	-0.117	0.465	0.464	-0.185		
**IT 13**	0.55	0.176	-0.034	0.511		

EFA= Exploratory Factor Analysis; IT=Item; The items that reach the acceptable factor loading index (≥|0.40|) are highlighted in bold

Table S6. EFA with orthogonal rotation (varimax) and internal consistency of 3 factors solution in SZ

	Factors				
	**1**	**2**	3	Cronbach's alpha	McDonald's omega
**IT 13**	**0.724**	0.111	-0.051		
**IT 9**	**0.615**	0.197	-0.105		
**IT 7**	**0.611**	-0.276	0.156	0.624	0.618
**IT 10**	**0.582**	-0.069	0.301		
**IT 2**	**0.55**	-0.059	-0.176		
**IT 12**	0.108	**0.751**	-0.159		
**IT 8**	-0.149	**0.721**	0.018	0.521	0.506
**IT 5**	-0.072	**0.47**	0.303		
**IT 14**	0.183	**0.455**	0.116		
**IT 1**	0.02	-0.048	**0.707**		
**IT 4**	-0.076	0.098	**0.628**	0.509	0.487
**IT 11**	0.317	0.106	**0.516**		
**IT 3**	-0.028	0.038	**0.489**		
**IT 6**	-0.181	0.491	0.453		
**IT 15**	-0.004	0.19	0.204		

EFA= Exploratory Factor Analysis; IT=Item; The items that reach the acceptable factor loading index (≥|0.40|) are highlighted in bold

Table S7. EFA with orthogonal rotation (varimax) and internal consistency of 2 factors solution in SZ

	Factors			
	1	2	Cronbach's alpha	McDonald's omega
**IT 6**	**0.671**	-0.169		
**IT 4**	**0.51**	0.011		
**IT 5**	**0.549**	-0.084		
**IT 8**	**0.531**	-0.234	0.610	0.607
**IT 1**	**0.458**	0.135		
**IT 11**	**0.43**	0.377		
**IT 12**	**0.425**	-0.014		
**IT 14**	**0.404**	0.14		
**IT 13**	0.031	**0.687**		
**IT 7**	-0.099	**0.657**		
**IT 10**	0.15	**0.626**	0.624	0.618
**IT 9**	0.056	**0.562**		
**IT 2**	-0.174	**0.52**		
**IT 3**	0.369	0.043		
**IT 15**	0.279	0.004		

EFA= Exploratory Factor Analysis; IT=Item; The items that reach the acceptable factor loading index (≥|0.40|) are highlighted in bold

Table S8. EFA with orthogonal rotation (varimax) and internal consistency of 4 factors solution in GP

	Factors					
	1	2	3	4	Cronbach's alpha	McDonald's omega
**IT 5**	**0.631**	-0.027	-0.076	-0.039	0.5	0.414
**IT 4**	**0.58**	0.034	-0.11	-0.053		
**IT 6**	**0.574**	-0.166	0.052	-0.115		
**IT 1**	**0.5**	-0.112	0.029	0.087		
**IT 15**	**0.435**	0.011	0.086	0.169		
**IT 7**	0.012	**0.598**	-0.112	0.047	0.641	0.636
**IT 13**	-0.23	**0.561**	0.186	-0.038		
**IT 2**	-0.211	**0.547**	0.005	-0.167		
**IT 9**	-0.015	**0.478**	0.124	0.157		
**IT 10**	0.111	**0.41**	-0.239	0.242		
**IT 14**	0.021	0.055	**0.653**	0.047	0.526	0.511
**IT 12**	-0.059	0.064	**0.572**	0.002		
**IT 8**	0.287	-0.149	**0.345**	-0.011		
**IT 11**	0.242	0.268	-0.176	**0.479**	**-**	**-**
**IT 3**	0.142	0.046	-0.178	-0.302		

EFA= Exploratory Factor Analysis; IT=Item; The items that reach the acceptable factor loading index (≥|0.40|) are highlighted in bold

Table S9. EFA with orthogonal rotation (varimax) and internal consistency of 3 factors solution in GP

	Factors				
	1	2	3	Cronbach's alpha	McDonald's omega
**IT 13**	**0.689**	-0.006	-0.128	0.666	0.667
**IT 10**	**0.646**	0.062	-0.166		
**IT 5**	**0.641**	-0.197	0.02		
**IT 2**	**0.625**	-0.076	0.013		
**IT 15**	**0.576**	0.096	0.115		
**IT 8**	-0.015	**0.702**	-0.088	0.630	0.628
**IT 14**	0.004	**0.62**	0.228		
**IT 11**	-0.279	**0.589**	0.282		
**IT 1**	0.14	**0.587**	-0.273		
**IT 7**	-0.29	**0.562**	0.063		
**IT 3**	0.327	**0.465**	-0.192		
**IT 6**	0.064	0.025	**0.767**	0.526	0.511
**IT 4**	-0.033	0.028	**0.737**		
**IT 12**	0.386	-0.195	**0.459**		
**IT 9**	0.103	-0.02	-0.288		

EFA= Exploratory Factor Analysis; IT=Item; The items that reach the acceptable factor loading index (≥|0.40|) are highlighted in bold

Table S10. EFA with orthogonal rotation (varimax) and internal consistency of 2 factors solution in GP

	**Factors**			
	1	2	Cronbach's alpha	McDonald's omega
**IT 5**	**0.625**	-0.069		
**IT 4**	**0.579**	-0.003		
**IT 6**	**0.526**	-0.231	0.666	0.667
**IT 1**	**0.499**	-0.129		
**IT 15**	**0.431**	0.001		
**IT 7**	0.052	**0.62**		
**IT 13**	-0.244	**0.501**		
**IT 2**	-0.217	**0.488**	0.641	0.636
**IT 9**	-0.005	**0.469**		
**IT 10**	0.184	**0.469**		
**IT 12**	-0.126	-0.014		
**IT 3**	0.115	-0.006		
**IT 14**	-0.062	-0.028		
**IT 11**	0.303	0.33		
**IT 8**	0.207	-0.209		

EFA= Exploratory Factor Analysis; IT=Item; The items that reach the acceptable factor loading index (≥|0.40|) are highlighted in bold

Figure S1. *EFAs path diagram of BCIS in SZ*

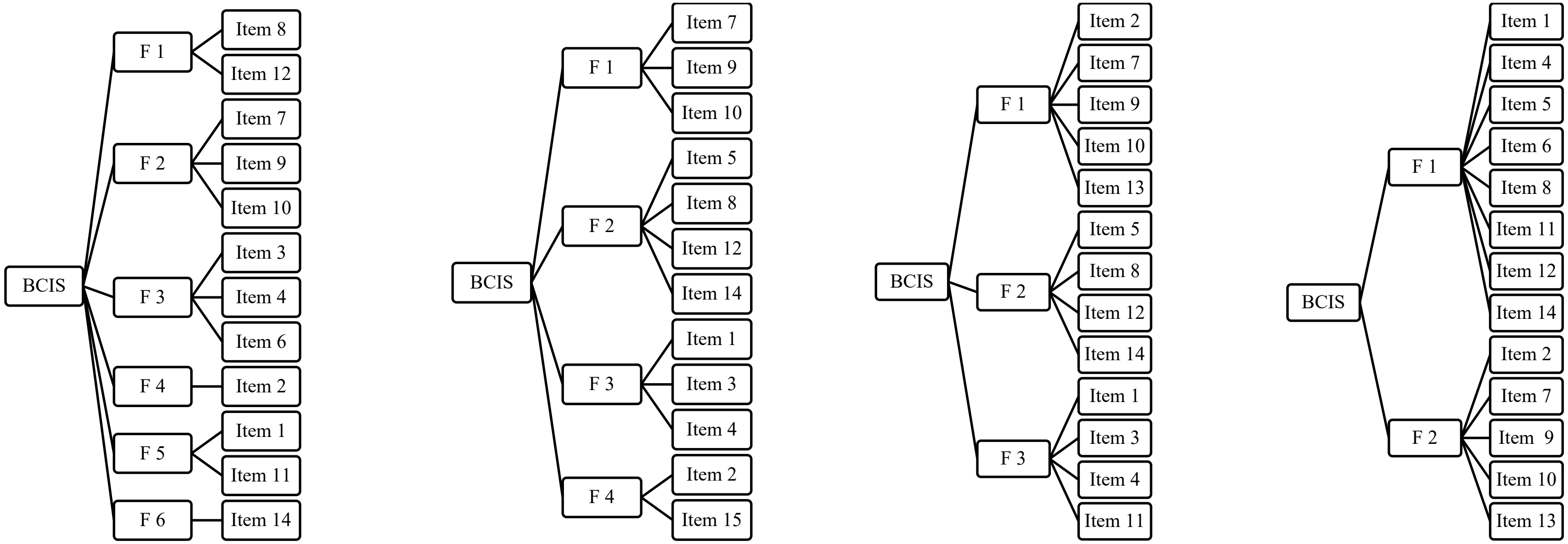

BCIS=Beck cognitive insight scale; F=Factor; SZ= Patients with schizophrenia

Figure S2. *EFAs path diagram of BCIS in GP*

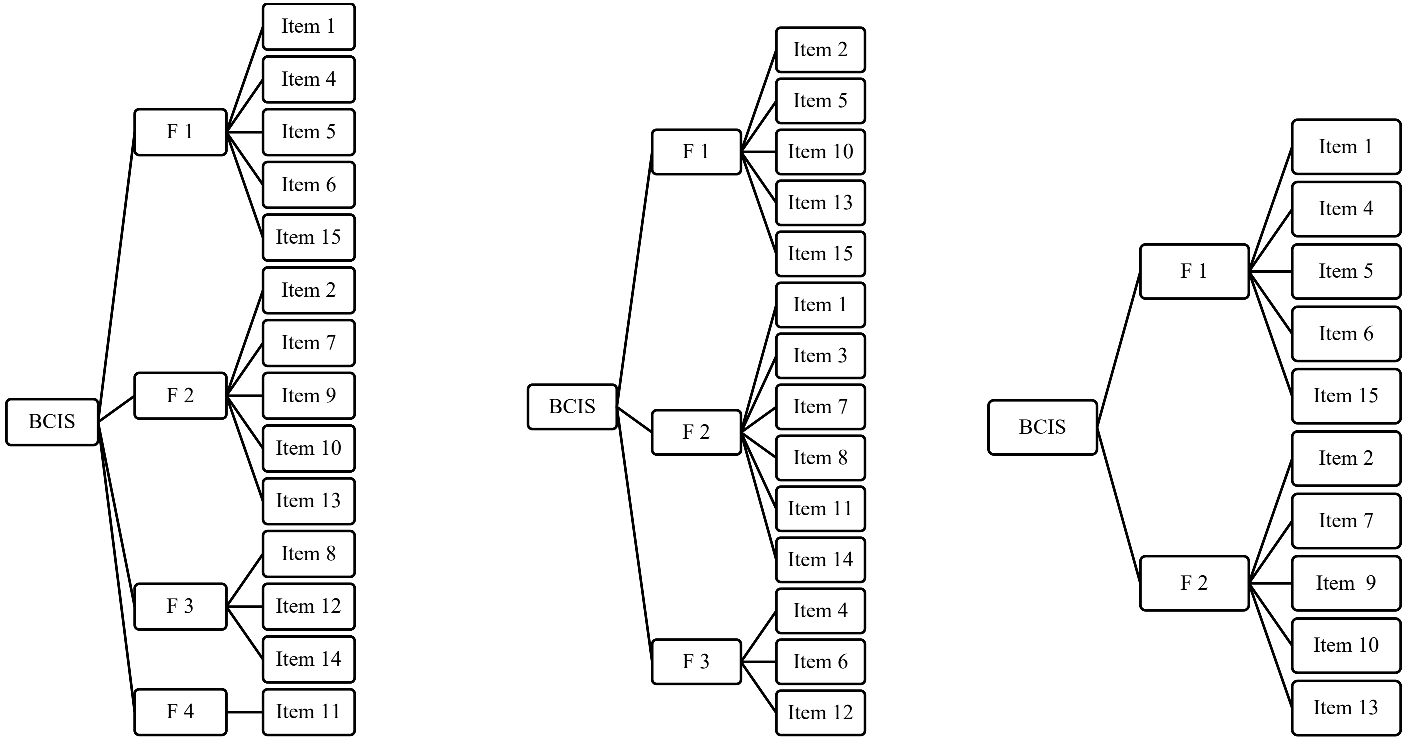

BCIS=Beck cognitive insight scale; F=Factor: GP= General population
